# Preconditioning of Spatial and Auditory Cues: Roles of the Hippocampus, Frontal Cortex, and Cue-Directed Attention

**DOI:** 10.3390/brainsci6040063

**Published:** 2016-12-19

**Authors:** Andrew C. Talk, Katrina L. Grasby, Tim Rawson, Jane L. Ebejer

**Affiliations:** Discipline of Psychology, School of Behavioural, Cognitive, and Social Sciences, University of New England, Armidale, NSW 2351, Australia; kgrasby@gmail.com (K.L.G.); t.rawson@centacarenenw.com.au (T.R.); janeebejer@gmail.com (J.L.E.)

**Keywords:** sensory preconditioning, source memory, spatial learning, episodic memory

## Abstract

Loss of function of the hippocampus or frontal cortex is associated with reduced performance on memory tasks, in which subjects are incidentally exposed to cues at specific places in the environment and are subsequently asked to recollect the location at which the cue was experienced. Here, we examined the roles of the rodent hippocampus and frontal cortex in cue-directed attention during encoding of memory for the location of a single incidentally experienced cue. During a spatial sensory preconditioning task, rats explored an elevated platform while an auditory cue was incidentally presented at one corner. The opposite corner acted as an unpaired control location. The rats demonstrated recollection of location by avoiding the paired corner after the auditory cue was in turn paired with shock. Damage to either the dorsal hippocampus or the frontal cortex impaired this memory ability. However, we also found that hippocampal lesions enhanced attention directed towards the cue during the encoding phase, while frontal cortical lesions reduced cue-directed attention. These results suggest that the deficit in spatial sensory preconditioning caused by frontal cortical damage may be mediated by inattention to the location of cues during the latent encoding phase, while deficits following hippocampal damage must be related to other mechanisms such as generation of neural plasticity.

## 1. Introduction

As organisms experience their environment, they may incidentally encode the location at which particular objects or events occur, and then retrieve that information later if it becomes useful. The ability of humans to latently encode and then retrieve details about the context or location in which an object or event was experienced has been referred to as source memory [1], and there has been interest in developing reduced or simplified procedures to test this memory capacity. Simplified models and, in particular, procedures in which memory can be assessed without collection of verbal responses, are necessary in functional imaging studies [2,3], and in studies of young children or animals [4].

In one common source memory procedure [3,5,6,7,8], subjects are asked to perform a task that requires them to attend to images of objects that are incidentally presented at different quadrants on a screen. Then, in a later test phase, the images are presented at the screen center and participants are asked to respond whether the object is new or previously seen (as a test of item recognition), and if it was previously seen, to indicate the original screen location (as a test of source memory recollection). This task has been used during functional imaging studies of source memory [2,3,7]. In other source memory studies, subjects have been asked to recollect in what color font recognized words were originally presented [9], whether recognized sentences were originally presented in a male or female voice [10], or where remembered trivia items were originally learned [11].

Activity in the hippocampus and the prefrontal cortex is correlated with both accurate encoding of source information and with later recollection of that information [2,3,7]. Reduced performance on source memory tests is a primary characteristic of age-related memory decline that begins progressing around age 30, while recognition of previously experienced cues is maintained until later in life [12]. Beginning at around age 30, increasing age is associated with reduced size and function of the prefrontal cortex; later in life, increasing age is associated with reduced size and function of the hippocampus [13,14]. As aging differentially affects memory abilities that depend on these structures, reduced source memory ability experienced earlier in life may be accounted for by reduced size and function of the frontal cortex, while increasing difficulty with cue recognition along with source memory deficit experienced later in life may be related to an additional hippocampal deficit [10,14]. Damage to the frontal cortex impairs attention to novel cues [15,16] and it has been suggested that source memory deficits associated with loss of frontal cortical function may be accounted for by reduced cue-directed attention [17]. Structures in the medial temporal lobe, such as the hippocampus, have also been implicated in cue-directed attention [18].

Through animal models of source memory, more precise information can be teased apart regarding which brain structures are involved in attention to environmental cues and with encoding of contextual information. Numerous types of tasks have been developed in which subjects learn the location at which a food reward was experienced, and where and when (or within which context) particular foods were experienced (reviewed by [19]). Rats have been shown to be able to remember source information about a chocolate reward by recalling whether contact with the chocolate was self-generated (by encountering chocolate while walking along a runway) or experimenter-generated (by being placed at the chocolate by an experimenter) [20]. There have also been tasks in which subjects remember the location at which neutral cues were incidentally experienced, or where and when neutral cues were experienced (reviewed by [21]).

The development of such studies in animals can be traced back to the classic studies of latent spatial learning by Tolman [22]. In one such study [23], rats explored a T-Maze that had food contained within differentiated, removable end-boxes. One of the end-boxes was then placed in another room, and the rats received an electric shock in it. When placed back on the maze, the rats reliably avoided the arm leading to the box in which they had been shocked. In order to complete this task, the rats must first latently encode the locations of the control and paired boxes. Then, during the test they must recall which box would be encountered with a left or right turn in order to avoid the box in which they had been shocked off-site.

We have used an analogous task involving single-trial discriminative sensory preconditioning of spatial and auditory cues. It is a rodent analog of minimized source memory procedures that involve incidental encoding and then recollection of the location at which sensory cues incidentally occurred, except that in human versions of the task, subjects are asked to latently encode and then recall the source locations of multiple cues [12]. The preconditioning task occurred as rats explored an elevated square platform, and involved a single pairing of the spatial cues associated with one corner and auditory cue. The opposite corner served as an unpaired control. The following day, we assessed recollection of information about the location at which the cue was delivered by pairing the auditory signal with a mild foot shock and then assessing avoidance of its former spatial location. Because the task occurs on an open table top, we can capture indexes of attention to the cue during the source memory encoding phase by looking for cue-directed behaviors. Then, we can independently assess recollection of the cue location at test. Thus, we can determine whether any lesion-induced deficit in source memory may be related to reduced attention to the cue, or whether deficits in other processes must be occurring such as in storage of information in the brain.

We have demonstrated that lesion of the medial prefrontal cortex (mPFC) impairs performance in this spatial sensory preconditioning task [24,25]. Here, we assessed any role for the dorsal hippocampus. Moreover, we assessed cue-directed orientation behaviors of subjects during the encoding phase to determine whether reduction of cue-directed attention may play a role in source memory deficits following either frontal cortical or hippocampal damage.

## 2. Methods

### 2.1. Dorsal Hippocampal Lesions

Forty male albino Wistar rats (330–550 g) were anaesthetized with Na-pentobarbital (60 mg/kg, intraperitoneally (i.p.)) and given either bilateral excitotoxic lesions to the dorsal hippocampus or sham surgeries. The excitotoxin injection coordinates were 2.8 mm posterior and ±1.6 mm lateral to bregma, then 3.3 mm ventral to dura, and 4.2 mm posterior and ±2.6 mm lateral to bregma, then 3.0 mm ventral to dura. The lesioning agent was a 0.4 µL volume of *n*-methyl-d-aspartate (NMDA) (20 µg/µL) in phosphate-buffered saline. Each injection was 80 s in duration, and the needle was left in place for an additional 220 s to allow diffusion. Twenty rats received infusions, and 20 control rats received identical surgeries except that the needle was held in place at each target site for 300 s with no infusion. Lignocaine HCl was injected alongside the incision at the time of surgery, and the rats received a nonsteroidal analgesic for two days after surgery. A two-week recovery period was given before behavioral procedures. All procedures were approved by the University of New England Animal Ethics Committee.

### 2.2. Preconditioning of Spatial and Auditory Cues

The subjects were first habituated to a fear conditioning chamber 20 min a day on three consecutive mornings to limit development of an association between the chamber context and shock unconditioned stimulus during the later conditioning phase. On the afternoon of day 3 (4 h after the third habituation session), the subjects were allowed to explore a 90 × 90 cm elevated black glass tabletop in a 2.5 × 3 m training room for 200 s. The room was indirectly illuminated by an 11 W florescent floor lamp, a string of light-emitting diodes, and an electroluminescent nightlight placed along the walls along with other spatial cues. A speaker (4 Ω) was placed 55 cm off each corner of the platform, and a camera was mounted directly above the platform. On the afternoon of day 4, the rats were allowed to explore the elevated platform for 400 s during a preconditioning session. During this time, an auditory cue (a train of 59 dB, 80 ms white noise pulses plus 20 ms onset and offset ramps, repeated at 2 Hz) was played from one of the speakers, beginning 200 s into the session and continuing for 100 s. The location of the active speaker was randomly assigned for each subject in a counterbalanced manner, so that each subject received a corner paired with the cue and an opposite unpaired control corner in a within-subjects design.

On the morning of day 5, the subjects were returned to the conditioning chambers for a fear conditioning session. Five trials were administered, separated by 8 min intervals. The conditioned stimulus was the same as during the preconditioning session at 59 dB inside the chamber, terminating with the onset of a 1 s, 1.0 mA foot shock. Four hours after this conditioning session, the subjects were returned to the elevated platform and allowed to freely explore for 400 s. The first 200 s of this period was without programmed stimulation, and then the rats were allowed to continue exploring while the auditory cue was played from the original corresponding speaker for an additional 200 s. The behavior of the subjects on the platform was recorded with the overhead camera at one frame per second.

### 2.3. Analysis of Video Recordings

We developed two scripts in Matlab (the MathWorks Inc., Natick, MA, USA) to analyze the video recordings. One script automatically tracked the coordinates of the center of the subject’s body in each frame of the recording. From these coordinates, we calculated the distance between the subject and the corner of the platform nearest the active speaker, and also detected periods of immobility which we defined as periods in which the subject did not move more than 0.4 cm between two frames. The other script was used to analyze head orientation with reference to the active speaker. An experimenter (blind to group assignments) used mouse input to indicate the location of the active speaker, and then, for every oddly-numbered frame (i.e., every 2 s in the recording), to indicate the location of the nose tip and the area directly between the subject’s ears. Head angle relative to the active speaker location was then automatically calculated. If the subject was grooming, or the head angle was otherwise not visible, then that was indicated by the user, and data from those frames was not used for generation of the averaged head orientation.

We also analyzed archived video recordings of the sensory preconditioning session of an experiment conducted by Rawson et al. [25] to determine head orientation to the cue of subjects with a lesion to the mPFC. In that study, we infused 0.07 M quinolinic acid in phosphate-buffered saline into two sites on each side of the frontal cortex. These sites were 3.5 mm anterior and 0.6 mm lateral to bregma, then 5.2 mm ventral to dura, and 2.5 mm anterior and 0.6 mm lateral to bregma, then 5.0 mm ventral to dura. The experiment conducted by Rawson et al. [25] used the same behavioral procedures as the current study.

### 2.4. Histology

The rats were deeply anaesthetized with Na-pentobarbital and perfused transcardially with physiological saline, followed by 10% formal saline. The brains were removed and placed into a sucrose–formalin solution for a minimum of six days. Using a freezing microtome, 50 µm coronal sections were cut and plated onto microscope slides for staining with cresyl violet. Digital images of coronal sections at 2.4 mm, 3.12 mm, 3.84 mm, and 4.56 mm posterior to bregma were collected. Lesion extent was indexed by an experimenter blind to subject performance, with reference to surviving hippocampal neural tissue in a control subject. This was done by using the image thresholding function of Adobe Photoshop to obtain a count of pixels in the surviving hippocampal cell fields of a sham-lesioned subject and comparing that with the number of pixels in the surviving neural tissue in the hippocampus of lesioned subjects.

## 3. Results

### 3.1. Hippocampal Lesion

**Figure 1:**
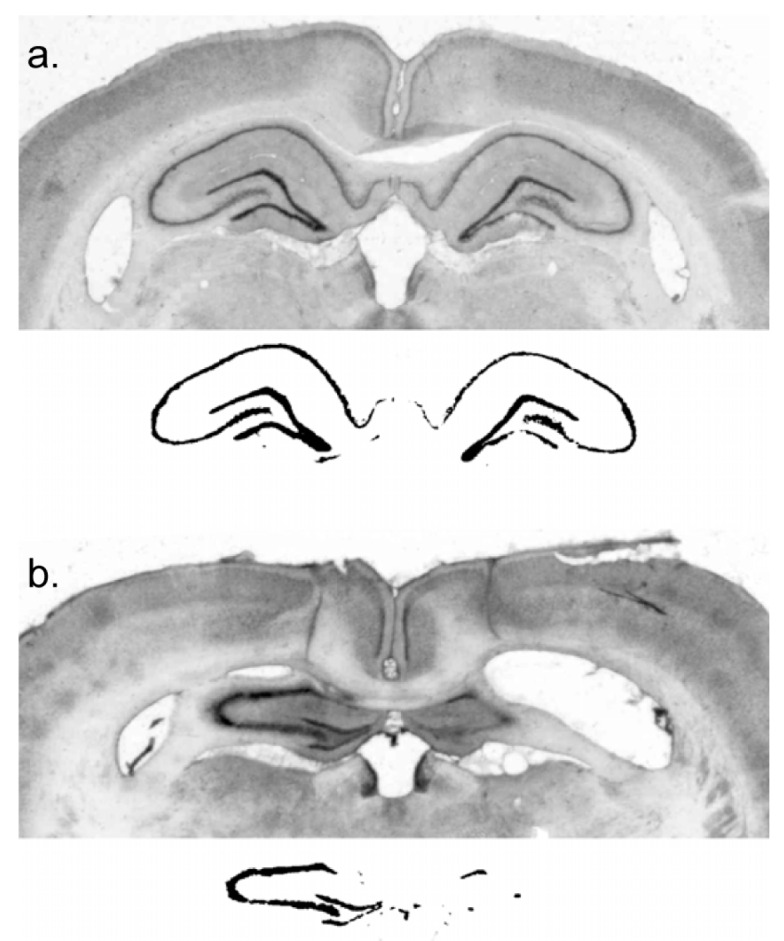
An index of lesion in each subject was obtained when digital images of the dorsal hippocampus were subjected to a grayscale threshold so that the area within the cell body layers of the surviving dorsal hippocampus was calculated. (**a**) Grayscale and thresholded images of a control subject; (**b**) images of a lesioned subject.

The residual dorsal hippocampus size of the lesioned subjects was characterized as 20.0% ± 3.6% (mean ± standard error) of sham and ranged from 0% to 55% of sham-treated subjects (Figure 1).

### 3.2. Hippocampal Lesions and Cue-Directed Attention

**Figure 2:**
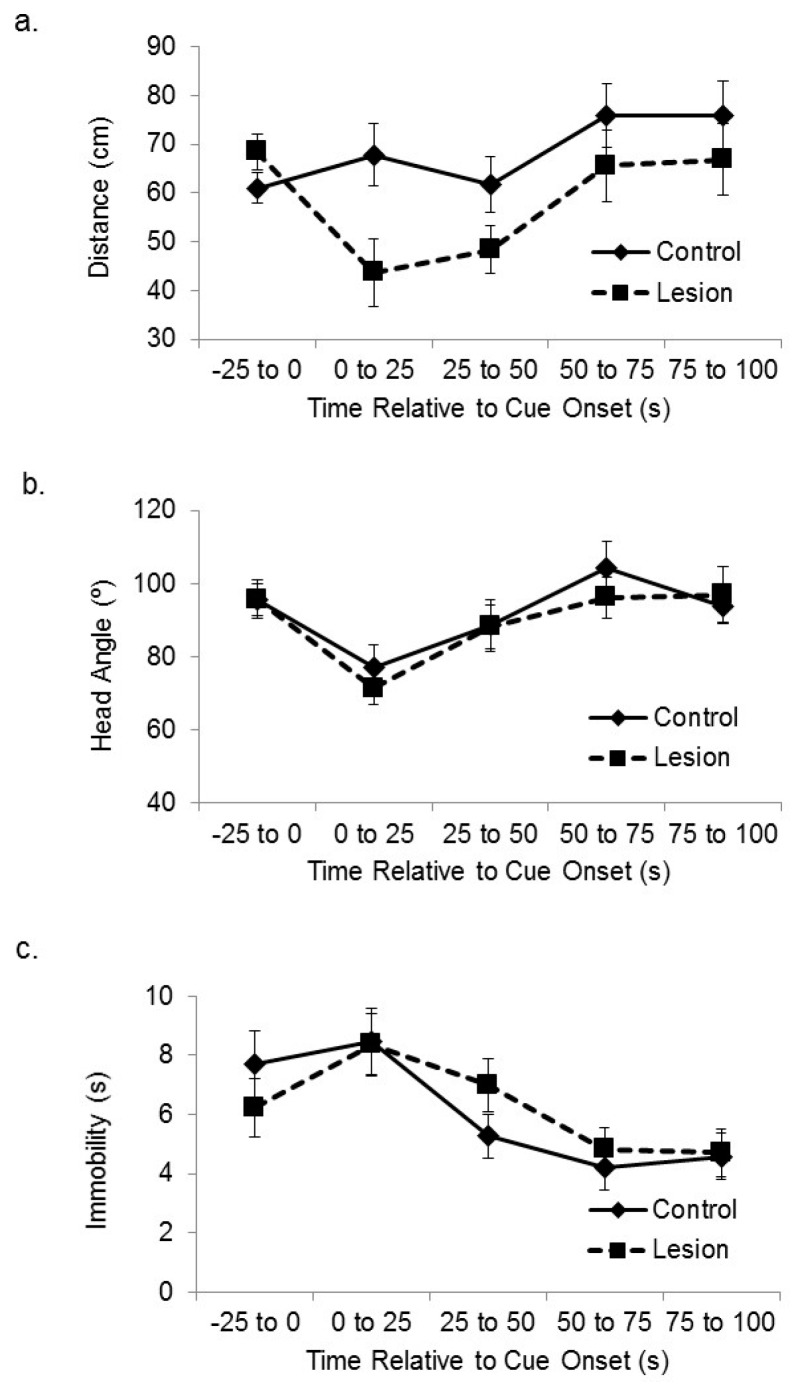
Hippocampus and cue-directed attention. (**a**) The distance between the subjects and the corner of the platform nearest the active speaker is plotted during the sensory preconditioning session, 100 s exposure to the auditory cue, and the immediately preceding baseline period. Subjects with lesions to the dorsal hippocampus approached the active speaker at cue outset more than control subjects; (**b**) The head angle relative to the location of the active speaker during the baseline period and cue exposure is plotted. There was no difference between groups in head orientation change towards the location of the auditory cue; (**c**) The number of seconds spent immobile during the baseline period and after cue onset is plotted. Subjects with lesions to the dorsal hippocampus were immobile for the same amount of time as controls. There was an effect of time after cue onset in which there was greater immobility soon after cue onset than in later periods.

Subjects with hippocampal lesions displayed greater cue-directed responses during exposure to the novel cue, in that they approached the active speaker more than control subjects when the auditory cue was initially presented in the encoding phase. Figure 2a shows the distance between the lesioned and control-treated subjects and the corner of the platform nearest the active speaker at baseline and at sequential 25 s time bins after cue onset. A mixed-design analysis of variance (ANOVA) on the distance between the subjects and the target corner in the sequential 25 s time blocks after cue onset revealed a significant effect of treatment group (F(1,35) = 5.46, *p* < 0.03), in which the lesioned rats approached the cue more than the sham control subjects. There was also a significant main effect of time after cue onset (F(3,105) = 4.87, *p* < 0.01). Increased attention to the cue by lesioned subjects was not seen in head direction responses. Subjects with hippocampal lesions expressed cue-elicited head direction responses towards the location of the active speaker that were similar in size to control subjects (Figure 2b). A mixed-design ANOVA on head angle relative to the cue for sequential 25 s time bins after cue onset showed a significant main effect of sequential time bin (F(3,105) = 5.81, *p* < 0.01), but no significant effect of group (F(1,35) = 0.23, ns), or interaction between time and group (F(3,105) = 0.29, ns). We also looked at amount of time spent immobile (Figure 2c). A mixed-design ANOVA on number of seconds spent immobile for sequential 25 s time bins after cue onset showed a significant main effect of sequential time bin (F(3,105) = 11.52, *p* < 0.01), but no significant effect of group (F(1,35) = 0.44, ns), or interaction between time and group (F(3,105) = 0.41, ns).

Due to the increased approach to the cue by subjects in the lesioned group than controls at the outset of cue exposure, we assessed average distance to the target corner in the two groups across the habituation and pre-exposure sessions. Rats have been shown to direct greater exploration to novel than familiar locations in mazes [26], and greater cue-elicited exploration of the area of the platform near the cue could account for any observed avoidance of that corner at the test phase in our study. However, there was no difference between groups in the overall distance to the target corner when averaged across the 600 s total time of the habituation and preconditioning sessions (t(35) = 0.88, ns). The lesioned group was on average 67.7 ± 2.5 cm from the target corner during these sessions, while the control group was 70.3 ± 1.7 cm from the corner. We also assessed baseline locomotion speed (i.e., before any stimulus delivery during the initial 200 s of habituation to the platform on day 3). The lesioned and control rats travelled 4.3 ± 0.59 and 4.9 ± 0.43 cm/s, respectively, and there was no significant difference in generalized locomotion between the groups (t(34) = 0.95, ns).

### 3.3. Hippocampal Lesions and Memory for Cue Location

**Figure 3:**
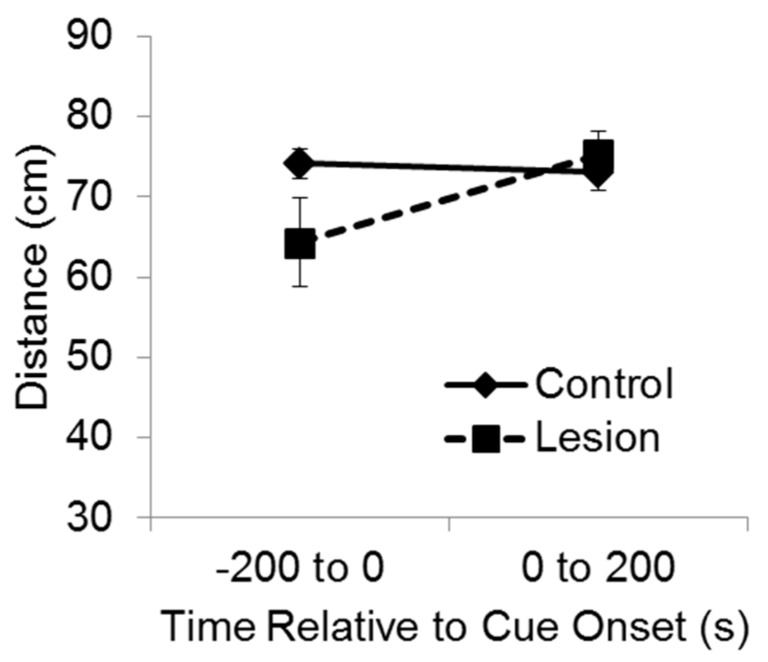
Hippocampus and recollection of cue location. The distance between the subjects and the corner of the platform nearest the active speaker is plotted during the test session. The lesioned subjects avoided the corner near the active speaker less than control subjects during baseline. After cue onset, the lesioned subjects moved away from the cue and avoided the corner near the active speaker the same as control subjects.

For purposes of analysis, the test session was divided into two phases, consisting of the 200 s baseline period and then 200 s during delivery of the cue (Figure 3). A mixed-design ANOVA indicated a significant interaction between group and test phase on the distance between the subject and the target corner (F(1,35) = 7.11, *p* < 0.02). Bonferroni’s t comparisons showed that before the cue was exposed to the subjects at test, the control subjects were significantly farther from the target corner compared to the lesioned subjects (*p* < 0.05), indicating greater recall and avoidance of that location. However, after the cue came on, the lesioned group moved so that they were significantly farther from the target corner than they were before cue onset (*p* < 0.05), and there was no significant difference between groups in distance from the target corner while the cue was playing. These results indicate that during the test baseline period, rats with hippocampal lesions failed to recollect and avoid the location at which the cue had been experienced the previous day. Yet, rats with hippocampal lesions were able to avoid the cue itself when it was presented at test, indicating they were capable of avoidance responding.

Because we found a high variance in the extent of dorsal hippocampal lesions (from 0% to 55% remaining hippocampal cell fields in the lesioned subjects), we were able to correlate extent of surviving hippocampus to performance in this task. A Pearson correlation between percent surviving hippocampus and distance from the target corner during the test before cue onset was not statistically significant (*r* = −0.29, ns), suggesting similar efficacy of the lesions in producing the source memory deficit regardless of size.

### 3.4. Medial Prefrontal Cortical Lesions and Cue-Directed Attention

**Figure 4:**
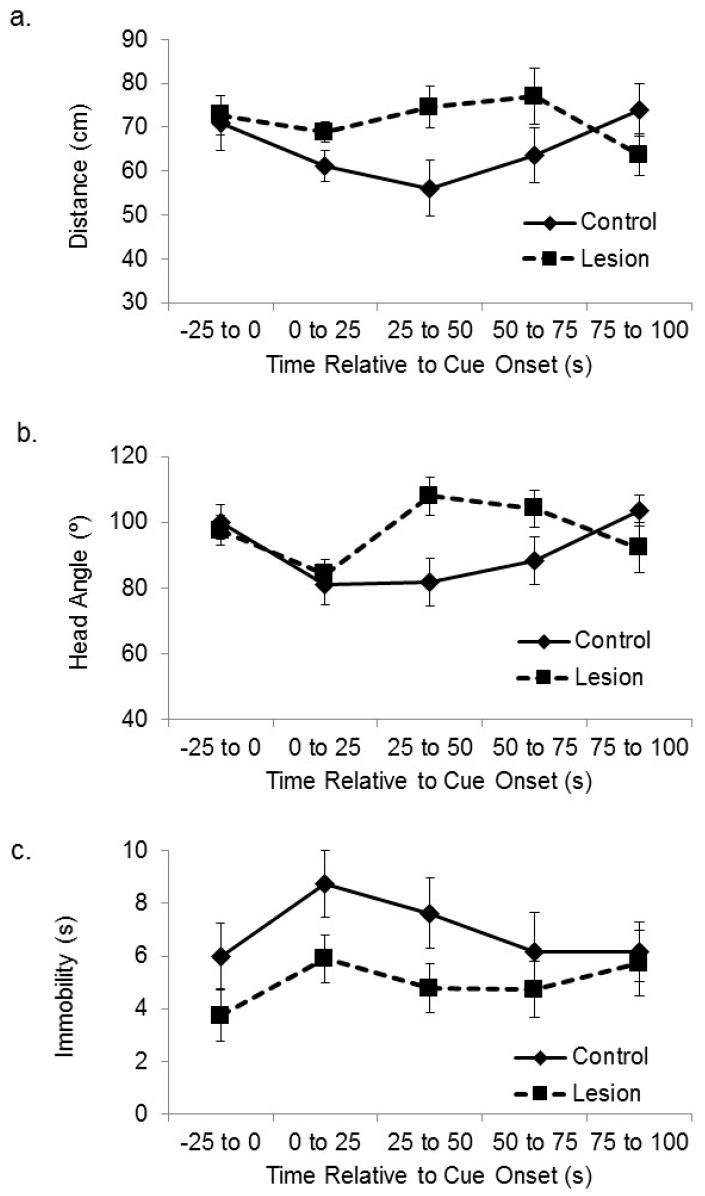
Medial prefrontal cortex (mPFC) and cue-directed orientation. (**a**) The distance between the subjects and the corner of the platform nearest the active speaker is plotted during the sensory preconditioning session exposure to the auditory cue and the immediately preceding baseline period. Control subjects approached the active speaker during the cue more than subjects with lesions to the mPFC; (**b**) The head angle relative to the location of the active speaker during the baseline period and cue exposure is plotted. Control subjects maintained head orientation towards the location of the auditory cue more than subjects with lesions to the mPFC; (**c**) The number of seconds spent immobile during the baseline period and after cue onset is plotted. The difference between the control and lesioned groups in seconds spent immobile was not statistically significant.

We have previously used these behavioral procedures to show that subjects with lesions to the medial prefrontal cortex are deficient at spatial sensory preconditioning [25]. Here, we have analyzed the video recordings collected during that study to assess whether those subjects were also deficient at cue-directed orientation during the preconditioning phase. Subjects with lesions to the mPFC expressed smaller responses to the location of the active speaker during the outset of initial exposure to cue (Figure 4), which is the opposite effect of hippocampal lesion. The subjects in the frontal cortical lesion group approached the active speaker less than control subjects when the auditory cue was initially presented, and they displayed less head orientation towards the active speaker. A mixed-design ANOVA on the distance between the subjects and the target corner in the sequential 25 s time blocks after cue onset revealed a significant interaction between treatment group and time (F(3,105) = 2.88, *p* < 0.05). Bonferroni’s t comparisons showed that the control subjects were significantly closer to the target corner compared to the lesioned subjects within the 1–25 s and 25–50 s time bins after stimulus onset (*p* < 0.05). A further mixed-design ANOVA on the angle between the head direction and the active speaker in the sequential 25 s time blocks after cue onset revealed a significant interaction between treatment group and time (F(3,105) = 4.13, *p* < 0.01). Bonferroni’s t comparisons showed that the control subjects oriented their head to the active speaker significantly more than the lesioned subjects during the 1–25 s and 25–50 s time bins after stimulus onset (*ps* < 0.05). A mixed-design ANOVA on number of seconds spent immobile during sequential 25 s time bins after cue onset showed no significant main effect of sequential time bin (F(3,105) = 2.13, ns), no significant effect of group (F(1,35) = 1.76, ns), or interaction between time and group (F(3,105) = 1.81, ns).

### 3.5. Control Group Comparisons

The two experiments were conducted at different times and treated as different studies in the statistical analysis above. However, they had similar behavioral and data analysis procedures. Here, we analyzed the control group data to explore whether the control group subjects in the two studies reacted differently to the cue while exploring the open platform. A mixed-design ANOVA on the distance between the control group subjects and the target corner in the sequential 25 s time blocks after cue onset revealed no significant effect of which study the data came from (F(1,37) = 1.38, ns), nor an interaction between study and time after cue onset (F(3,111) = 0.35, ns). A similar ANOVA on head direction in the sequential 25 s time blocks after cue onset revealed no significant effect of study (F(1,37) = 0.20, ns), nor an interaction between study and time after cue onset (F(3,111) = 0.05, ns). Finally, an ANOVA on time spent immobile in the sequential 25 s time blocks after cue onset revealed no significant effect of study (F(1,37) = 1.60, ns), nor an interaction between study and time after cue onset (F(3,111) = 0.56, ns). These results suggest there were only small, and not statistically significant, differences between the control group data in the two studies.

## 4. Discussion

The results indicate a dissociation in the functions of the hippocampus and medial prefrontal cortex during encoding of the source location of an auditory cue. Analysis of cue-directed movement during the memory encoding phase suggests that attention to the cue was enhanced in subjects with hippocampal lesions. A similar analysis of video recordings of rats with lesions to the mPFC indicated that lesioned subjects exhibited less cue-directed attention. These results provide further support for a dissociation of hippocampal and frontal cortical function during incidental memory for the location of cues within an environment. The frontal cortex is important for attention to novel cues and their spatial locations, while the hippocampus is not important for cue-directed attention but nonetheless may be critical for the rapid development of arbitrary associations between cues and locations and the storage of those associations in the brain [27]. It has been known for some time that lesions to the hippocampus do not attenuate the orienting response and, in fact, can reduce the amount of orienting response habituation displayed by rats [28]. These results, along with the current ones, point to a role for the hippocampus in generation and storage of associations, rather than attention, during latent learning about the locations of cues. Given the level of neural plasticity displayed by hippocampal neurons, we suggest that during latent learning about cue location, the cue–location association is initially stored in the hippocampus, while the frontal cortex is important for cue-directed attention.

The subjects experienced an auditory cue at a corner of an elevated platform during a period of free exploration. The cue was then paired with shock, and the subjects placed back on the platform in a test session. Control subjects avoided the corner that had been near the location of the cue more than subjects with damage to the dorsal hippocampus. Avoidance at the test baseline reflects recollection of the location at which the cue occurred during preconditioning. When the cue was then presented from the original corresponding speaker, lesioned subjects also began avoiding the target corner. Avoidance after cue onset at test assesses cue recognition and provides a control assuring that the subjects with hippocampal lesions were capable of avoidance performance. Less avoidance of the paired location during the test baseline period by the lesioned subjects must thus reflect a memory failure, rather than lesion-induced hyperactivity or other performance deficit.

### 4.1. The Hippocampus and Incidental Learning about the Location of Cues

The effect of hippocampal lesions on sensory preconditioning depends on the nature of the preconditioning procedure. For example, sensory preconditioning involving auditory and visual cues is sensitive to hippocampal damage [29,30,31], but sensory preconditioning of gustatory cues survives hippocampal lesion [32]. Iordanova et al. [33] conducted a discriminative sensory preconditioning task involving pairings of auditory cues with spatial contexts and showed that ability to form and express associations between a cue and spatial context can survive hippocampal lesion. Two contexts and two auditory cues were used, with each context paired with a cue across multiple days. Rats with hippocampal lesions were able to freeze more in the appropriate context after one of the two auditory cues was paired with shock, indicating that they encoded and retrieved the spatial sensory association. Yet, an effect of hippocampal lesion was observed after the element of time of day was introduced to the task; each auditory cue was paired with one context in the morning and with the other context in the afternoon. After one of the auditory cues was paired with shock, control rats froze in the appropriate context depending on the time of day the test was conducted. Rats with hippocampal lesions were unable to make this three-element (i.e., cue, context, and time of day) configural association (see also [34,35,36]).

Here, we observed an effect of hippocampal damage on cue-location preconditioning without a third configural element. It is possible that we obtained an effect of hippocampal lesion because of the single-trial nature of the current task or because subjects must encode and recall the specific location of the cue, rather than only its presence within a particular context. Given Iordanova et al. [33] used a limited number of contexts and cues, it is possible that the discrepancy in results can be explained by greater reliance on familiarity-based recognition in the previous study. The subjects in Iordanova et al. [33] and similar studies may create associations that are based on limited numbers of cues and contexts using familiarity memory, based on binding mechanisms [37] or associative familiarity [38]. In contrast, the hippocampus may be required to support tasks relying more on recollection of more elements (as in the three-way configural experiment by Iordanova et al. [33]) or recollection of multiple (e.g., more locations as in the current study) associates. This interpretation is consistent with the role of the hippocampus in recollection but not in familiarity. One trial learning about the location of food reinforcement requires a functional hippocampus in rats [39], and human subjects with hippocampal damage who are given incidental single exposures to cues at specific locations in the environment are subsequently unable to recall details about those locations [10,40].

### 4.2. Attention and Learning

The sensory preconditioning task we used is similar to source memory procedures in which human subjects are incidentally exposed to cues in an environment, and then later assessed on whether they can recollect the location or details about the context in which the cues were experienced (e.g., [3,7]). For example, Cansino [12] presented pictures of objects at various quadrants on a screen and asked subjects to respond whether the objects were natural or artificial. Then, in a surprise recall test, the subjects were presented with some of the objects at the screen center, along with some new objects, and asked whether they have seen the object before, and if so, to indicate the original location. Both hippocampal and prefrontal cortical activation are related to accurate induction and recollection of the screen location of cues, while cue recognition is not associated with such activity [2,3,7].

Evidence we reported here and previous evidence suggest that the effect of frontal cortex damage on source memory can be accounted for by reduced attention to cues. In both rodents and humans, damage to the frontal cortex leads to smaller orienting responses to novel cues [15,16,41], and source memory impairment related to impaired frontal cortical function can be eliminated through instructions to specifically attend to the context of the cue during induction [17]. This suggests that subjects with frontal cortex damage can recall source information as long as sufficient attention to the cues and their sources occurred during encoding.

The medial temporal lobe has also been shown to mediate attention, in that human subjects with pathology that includes the hippocampus, parahippocampal gyrus, and the entorhinal cortex showed a loss of reactivity to novel stimuli in event-related potentials [18]. However, it has also been shown in rodents that lesions restricted to the hippocampus can enhance or prolong orienting responses to novel cues [28]. One hypothesis regarding attention and hippocampal function has been that attentional networks, such as those which occur in the mPFC, can lead to increased stability of hippocampal cell place fields and spatial task performance [42,43]. The hippocampus sends strong projections to the mPFC [44], but the reciprocal connection from the mPFC to the hippocampus is indirect. There is a large indirect connection via the nucleus reuniens of the thalamus [45], and the mPFC also has a smaller level of projection to the entorhinal cortex [46]. The results of the current study largely support models of hippocampal and frontal cortical functioning that suggest the hippocampus may not itself be essential for attention to the location of novel cues, but is essential for subsequent memory of their locations in the environment, while the frontal cortex is important for cue-directed attention and, consequently, for the encoding of cue location by the hippocampus.

## 5. Conclusions

We have shown a dissociation in the functions of the hippocampus and medial prefrontal cortex during encoding of the source location of an auditory cue by rats. Rats with hippocampal lesions displayed greater cue-directed behavior than control rats during the memory encoding phase, while rats with lesions to the mPFC displayed less cue-directed behavior than controls. These results support theories that suggest different functions for the hippocampus and frontal cortex during incidental memory about the location of environmental cues. The frontal cortex seems to be important for attention to cues and their surrounding contextual information, while the hippocampus is not important for cue-directed attention. The hippocampus may instead be important for rapid development of arbitrary associations between discrete cues and contextual information and be a storage location of those associations in the brain.

## References

[1] Shimamura A.P., Squire L.R. (1987). A neuropsychological study of fact and source amnesia. J. Exp. Psychol. Learn. Mem. Cogn..

[2] Rugg M.D., Fletcher P.C., Chua P.M.L., Dolan R.J. (1999). The role of the prefrontal cortex in recognition memory and memory for source: An fMRI study. Neuroimage.

[3] Cansino S., Maquet P., Dolan R.J., Rugg M.D. (2002). Brain activity underlying encoding and retrieval of source memory. Cereb. Cortex.

[4] Clayton N.S., Russell J. (2009). Looking for episodic memory in animals and young children: Prospects for a new minimalism. Neuropsychologia.

[5] Cansino S., Trejo-Morales P., Estrada-Manilla C., Pasaye-Alcaraz E.H., Aguilar-Castaneda E., Salgado-Lujambio P., Sosa-Ortiz A.L. (2015). Brain activity during source memory retrieval in young, middle-aged and old adults. Brain Res..

[6] Cansino S., Hernandez-Ramos E., Trejo-Morales P. (2012). Neural correlates of source memory retrieval in young, middle-aged and elderly adults. Biol. Psychol..

[7] Slotnick S.D., Moo L.R., Segal J.B., Hart J. (2003). Distinct prefrontal cortex activity associated with item memory and source memory for visual shapes. Brain Res. Cogn. Brain Res..

[8] Mawdsley M., Grasby K., Talk A. (2014). The effect of sleep on item recognition and source memory recollection among shift-workers and permanent day-workers. J. Sleep Res..

[9] Doerksen S., Shimamura A.P. (2001). Source memory enhancement for emotional words. Emotion.

[10] Glisky E.L., Polster M.R., Routhieaux B.C. (1995). Double dissociation between item and source memory. Neuropsychology.

[11] Janowsky J.S., Shimamura A.P., Squire L.R. (1989). Source memory impairment in patients with frontal-lobe lesions. Neuropsychologia.

[12] Cansino S. (2009). Episodic memory decay along the adult lifespan: A review of behavioral and neurophysiological evidence. Int. J. Psychophysiol..

[13] Bartzokis G., Beckson M., Lu P.H., Nuechterlein K.H., Edwards N., Mintz J. (2001). Age-related changes in frontal and temporal lobe volumes in men: A magnetic resonance imaging study. Arch. Gen. Psychiatry.

[14] Schacter D.L., Savage C.R., Alpert N.M., Rauch S.L., Albert M.S. (1996). The role of hippocampus and frontal cortex in age-related memory changes: A PET study. Neuroreport.

[15] Knight R.T. (1984). Decreased response to novel stimuli after prefrontal lesions in man. Electroencephalogr. Clin. Neurophysiol..

[16] Daffner K.R., Mesulam M.M., Scinto L.F.M., Acar D., Calvo V., Faust R., Chabrerie A., Kennedy B., Holcomb P. (2000). The central role of the prefrontal cortex in directing attention to novel events. Brain.

[17] Glisky E.L., Rubin S.R., Davidson P.S.R. (2001). Source memory in older adults: An encoding or retrieval problem?. J. Exp. Psychol. Learn. Mem. Cogn..

[18] Knight R.T. (1996). Contribution of human hippocampal region to novelty detection. Nature.

[19] Allen T.A., Fortin N.J. (2013). The evolution of episodic memory. Proc. Natl. Acad. Sci. USA.

[20] Crystal J.D., Alford W.T., Zhou W., Hohmann A.G. (2013). Source memory in the rat. Curr. Biol..

[21] Robertson B.A., Eacott M.J., Easton A. (2015). Putting memory in context: Dissociating memories by distinguishing the nature of context. Behav. Brain Res..

[22] Tolman E.C. (1948). Cognitive maps in rats and men. Psychol. Rev..

[23] Tolman E.C., Gleitman H. (1949). Studies in learning and motivation; equal reinforcements in both end-boxes; followed by shock in one end-box. J. Exp. Psychol..

[24] Parnell R., Grasby K., Talk A. (2012). The prefrontal cortex is required for incidental encoding but not recollection of source information in rodents. Behav. Brain Res..

[25] Rawson T., O’Kane M., Talk A. (2010). The medial prefrontal cortex and memory of cue location in the rat. Neurobiol. Learn. Mem..

[26] Hannesson D.K., Vacca G., Howland J.G., Phillips A.G. (2004). Medial prefrontal cortex is involved in spatial temporal order memory but not spatial recognition memory in tests relying on spontaneous exploration in rats. Behav. Brain Res..

[27] Rolls E.T. (2010). A computational theory of episodic memory formation in the hippocampus. Behav. Brain Res..

[28] Kaye H., Pearce J.M. (1987). Hippocampal lesions attenuate latent inhibition and the decline of the orienting response in rats. Q. J. Exp. Psychol. B.

[29] Port R.L., Murphy H.A., Magee R.A. (1996). Age-related impairment in instrumental conditioning is restricted to initial acquisition. Exp. Aging Res..

[30] Port R.L., Patterson M.M. (1984). Fimbrial lesions and sensory preconditioning. Behav. Neurosci..

[31] Talk A.C., Gandhi C.C., Matzel L.D. (2002). Hippocampal function during behaviorally silent associative learning: Dissociation of memory storage and expression. Hippocampus.

[32] Ward-Robinson J., Coutureau E., Good M., Honey R.C., Killcross A.S., Oswald C.J.P. (2001). Excitotoxic lesions of the hippocampus leave sensory preconditioning intact: Implications for models of hippocampal function. Behav. Neurosci..

[33] Iordanova M.D., Burnett D.J., Aggleton J.P., Good M., Honey R.C. (2009). The role of the hippocampus in mnemonic integration and retrieval: Complementary evidence from lesion and inactivation studies. Eur. J. Neurosci..

[34] Kesner R.P., Hunsaker M.R., Warthen M.W. (2008). The CA3 subregion of the hippocampus is critical for episodic memory processing by means of relational encoding in rats. Behav. Neurosci..

[35] Li J.S., Chao Y.S. (2008). Electrolytic lesions of dorsal CA3 impair episodic-like memory in rats. Neurobiol. Learn. Mem..

[36] Langston R.F., Stevenson C.H., Wilson C.L., Saunders I., Wood E.R. (2010). The role of hippocampal subregions in memory for stimulus associations. Behav. Brain Res..

[37] Yonelinas A.P., Aly M., Wang W.C., Koen J.D. (2010). Recollection and familiarity: Examining controversial assumptions and new directions. Hippocampus.

[38] Mayes A., Montaldi D., Migo E. (2007). Associative memory and the medial temporal lobes. Trends Cogn. Sci..

[39] Day M., Langston R., Morris R.G.M. (2003). Glutamate-receptor-mediated encoding and retrieval of paired-associate learning. Nature.

[40] Gold J.J., Smith C.N., Bayley P.J., Shrager Y., Brewer J.B., Stark C.E.L., Hopkins R.O., Squire L.R. (2006). Item memory, source memory, and the medial temporal lobe: Concordant findings from fMRI and memory-impaired patients. Proc. Natl. Acad. Sci. USA.

[41] Dias R., Honey R.C. (2002). Involvement of the rat medial prefrontal cortex in novelty detection. Behav. Neurosci..

[42] Kyd R.J., Bilkey D.K. (2003). Prefrontal cortex lesions modify the spatial properties of hippocampal place cells. Cereb. Cortex.

[43] Muzzio I.A., Kentros C., Kandel E. (2009). What is remembered? Role of attention on the encoding and retrieval of hippocampal representations. J. Physiol..

[44] Jay T.M., Glowinski J., Thierry A.M. (1989). Selectivity of the hippocampal projection to the prelimbic area of the prefrontal cortex in the rat. Brain Res..

[45] Vertes R.P. (2004). Differential projections of the infralimbic and prelimbic cortex in the rat. Synapse.

[46] Sesack S.R., Deutch A.Y., Roth R.H., Bunney B.S. (1989). Topographical organization of the efferent projections of the medial prefrontal cortex in the rat: An anterograde tract-tracing study with *Phaseolus vulgaris* leucoagglutinin. J. Comp. Neurol..

